# Determinants of incomplete penta vaccination among children aged 12 to 23 months in South-West Ethiopia

**DOI:** 10.1038/s41598-024-62153-5

**Published:** 2024-05-31

**Authors:** Bilisumamulifna Tefera Kefeni, Samuel Ejeta Chibsa, Gebiso Roba Debele

**Affiliations:** 1 Department of Public Health, College of Health Sciences, Mattu University, Mettu, Ethiopia; 2 Department of Midwifery, College of Health Sciences, Mattu University, Mettu, Ethiopia

**Keywords:** Determinants, Incomplete vaccination, 12–23 months’ children, South west, Ethiopia, Immunology, Medical research

## Abstract

Globally dropout rate for the three dose of penta (DPT) vaccine was highest in the African region. This mainly occurred in the African Region including Ethiopia. Despite high national incomplete vaccination status, there is lack of study on the determinants of incomplete vaccination in south west region, Ethiopia. Therefore, this study was conducted to identify determinants of incomplete Penta vaccination among children aged 12 to 23 months in Mettu district South-West Ethiopia. A Community based case–control study was conducted from April 24, May 23, 2022 in South-west Ethiopia. Data was collected from 297 participants (99 cases and 198controls) by using simple random sampling techniques**.** Cases were children age from 9 to 23 months who missed at least one dose from the routine vaccine and controls were completed the entire routine vaccine schedule. Data was entered to Epi-data version 3.1 and exported to SPSS version 23 for statistical analyses. Binary and multivariable logistic regression with a 95% CI and a p-value of < 0.05 was done to declare statistical significance. A total of 95 cases and 197 controls participated in the study. Rural residence [AOR: 3.9; 95% CI; (1.6**,** 9.4)], wealth indexes [AOR: 3.6; 95% CI; (1.8**,**7.0)], mothers unimmunized tetanus toxoid [AOR: 4.3; 95% CI; (2.1, 8.6)], postponed schedule [AOR: 4.6; 95% CI; (2.4**,** 8.8)], un satisfied to service [AOR: 3.7; 95% CI; (1.7,7.6)] and poor perception on benefit of vaccine [AOR:2.7; 95% CI; (1.2, 6.1)] were determinants of incomplete vaccination. Rural Residence, Family wealth index of poor; Mother not received tetanus vaccination; postponed vaccination schedule client satisfaction and caretaker perception on benefit of vaccination were identified determinants of incomplete vaccination.

Health information should be given for the community and child caretaker on the benefit of complete vaccination. Community should be encouraged to not post pond vaccine schedule. Pregnant women should be strengthening to receive tetanus toxoid vaccine during pregnancy.

## Introduction

Vaccine stimulate the body’s own immune system to protect the person against subsequent infection or disease^1^. Vaccinations are one of the most cost effective and impactful health interventions used across the world and have resulted in dramatic declines and regional elimination of many serious childhood infectious diseases^2^. Unlike many other health interventions, vaccines have both short and long-term benefits not only for individuals but also for the entire population^3^.

The proportion of children who started but did not complete a vaccination was a series indicator of immunization program performance, estimated to be 5% in 2016 for the 3-dose DTP series and with dropout highest in the African Region (11%) and lowest in the Western Pacific Region (0.4%)^4^. Globally 2.5 million children under five years of age die every year due to vaccine preventable diseases^5^ Although the world witnessed a tremendous reduction in child mortality between 1990 and 2015, sub-Saharan Africa (SSA) is still characterized by high under-five deaths^6^. One-quarter of these deaths are preventable through interventions such as immunization^7^. The major causes of under five deaths in Ethiopia are neonatal related with vaccine preventable like that of infection (47%), pneumonia (17%), diarrhea (8%) and measles (4%) with under nutrition associated with around half of the deaths^8^.

Ethiopian Demographic Health Survey (EDHS) 2019 report shows there is a 15 percentage-point dropout rate at the national level from the first to the third dose of PENTA (DPT-HepB-Hib vaccine) and 17 percent dropout rate from the first dose of PENTA (DPT-HepB-Hib vaccine) to the measles^9^. In addition 18 percentage-point dropout rate from the first to the third dose of polio vaccine, 19 percent dropout rate from the first dose of polio vaccine to measles and only 43.1 percent of the child had complete vaccination^9^.

EDHS 2019 mini survey report shows there is 11.3 percentage-point dropout rate at the Gambella region level from the first to the third dose of PENTA (DPT-HepB-Hib vaccine) and 18.7 percent dropout rate from the first dose of PENTA (DPT-HepB-Hib vaccine) to the measles^9^. Also 29.3 percentage dropout rate from the first to the third dose of polio vaccine, 27.4 percent dropout rate from the first dose of polio vaccine to measles and only 38.3 percent of the child had complete vaccination and camper to the national level there is high rate of incomplete vaccination at Gambella region^9^.

According to available literatures on incomplete penta vaccination was absence of postnatal care (PNC) follow-up, Parity of the mothers ≥ 3, knowledge about vaccination, Misconception on vaccine contraindication, and absence of Antenatal care (ANC) follow-up and home delivery were independent predictors of immunization^10^.

DPT3 coverage increased from 52% in 2003 to 87% in 2014 in coverage among regions, reaching every district strategic approach is recast to reaching every children strategic approach in order to deal with inequities within districts^11^. The country has mobilized women development armies or volunteers, health extension workers, and health facilities to deliver its immunization services^12^. Despite major progresses to improve the health status of the population in the last two decades, Ethiopia’s child population still face a high rate of morbidity and mortality and the health status remain low in terms of quality and equity in access to services^11^. Thus, based on this we conducted our research mainly focusedng on health service and Sociodemographic factors, because EDHS 2019 result showed that only 52.9 Percent of children in Oromia region are completely vaccinated the last dose of DPT3. Therefore, this study was employed to assess the determinant of incomplete Penta Vaccination among children aged 12 to 23 months at Illu Aba Bor mettu district, South-West Ethiopia.

## Materials and methods

### Study setting and design

A Community based case–control study was conducted at Illu Aba Bor, mettu district, south west Ethiopia from April 24-May 23, 2022. Mettu is located in Oromia Regional State at a distance of 600 km south-west of Addis Ababa (the capital city of Ethiopia). The total population was 40,483, of which 13,284 were females in the reproductive age group. In the town, there is 1 health center and 1 public hospital that provide maternal and child health services. Illu Aba Bor zone has total population of 32,660(16,003 males and 16,657 females).

### Source population and study population

All children aged 12 to 23 months who had started at least one immunization program in the study area were included in the source population. Children aged 12–23 months who had missed at least one dose of the required vaccine and not start immunization, as determined by the immunization registration book, were considered as cases. Children aged 12–23 months who had received all of the prescribed vaccines, as determined by the vaccination registration book, served as controls.

### Eligibility criteria

#### Inclusion criteria

All children who had a full address from EPI registration book and caretakers of children living in the study area at the time of the study for cases and controls.

#### Exclusion criteria

All Children whose mothers/caregivers who was unable to answer the questions due to any reason cases and controls. Children who have incomplete information about their address cases and controls were excluded. All children’s whose mothers or caretakers who changed their residence or the child was dead for cases and controls had been excluded.

### Sample size determination

Sample size was determined by stat calc Epi Info version 7 using determinants of incomplete immunization following assumptions were put into consideration: Confidence level of 95%, power of the study 80%, the case–control ratio 1:2, expected percept of exposures in control, expected percent exposure among cases taken from previous study^13^, and non-response rate of 10%. Required sample size calculated by considering absence of PNC follow-up was selected since it gives maximum sample size 45.9% among controls and 64.7% among cases^13^. Assuming 95% confidence interval, 80% power and control to case ratio of 2:1, the final sample size including 10% non-response rate is 297 (99 cases and 198 controls).

### Sampling procedure

**Figure 1:**
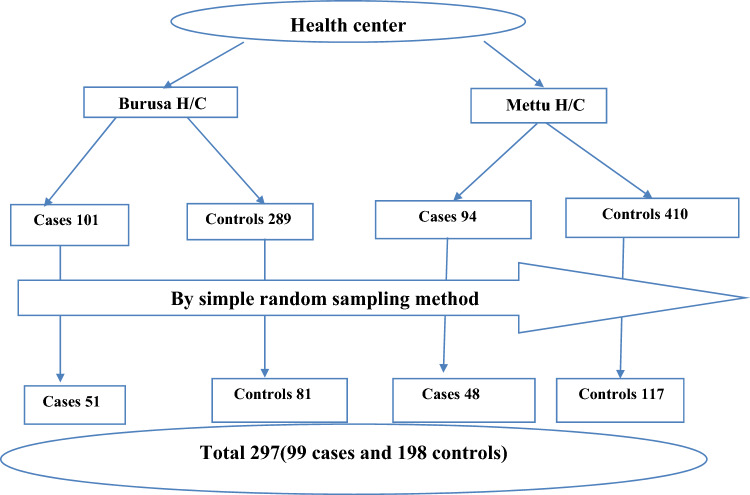
schematic presentation of sampling procedures used to assess the determinant of incomplete Penta Vaccination among children aged 12 to 23 months at Illu Aba Bor Mettu districts, South-West Ethiopia.

There are two health centers in the district. Sampling frame was developed separately for cases and controls in each health center from EPI registration. Data from health posts providing vaccination service was included in the catchment area of health centers. Proportionally allocated sample size of cases and controls from each health facility was selected by simple random sampling method by using computer generated random number. Cases and controls from health center were included in the respective catchment area health facilities. All children fitting case and control definition extracted from EPI register and sampled for the study was traced to their households by using health extension workers (HEWs). Total of Ninety-Nine (99) cases and total of one hundred ninety-eight controls (198) was selected for the study (Fig. 1).

### Operational and term definitions

#### Complete vaccination

A child between age range of 12–23 months who received all the routine vaccines like1 dose of BCG, 3 doses of pentavalent, 3 doses of polio, 3 doses of PCV, 2 doses of Rota vaccine and first dose of measles vaccine.

#### Incomplete vaccination

Occur when the child started vaccination and missed at least one of the routine vaccines before his/her 1^st^ birth day.

#### Perception about vaccine side effect

Respondents were asked six misperception questions on vaccine side effect by using likert scale. After computing the mean score for total constructs, greater than or equal to mean score had misperception on vaccine side effect and below means score had favorable perception. Cronbach’s Alpha for reliability test was 0.94.

##### Knowledge on the vaccine preventable disease

Respondents were asked about which vaccine preventable disease they knew and those who knew four or more had good knowledge and less than four had poor knowledge. Cronbach’s Alpha for reliability test was 0.84.

##### Knowledge on the schedule of vaccination

Mothers/care givers were asked four questions which were related to schedule of immunization and who knew three or more questions correctly had good knowledge and who knew less than three questions correctly had poor knowledge. Cronbach’s Alpha for reliability test was 0.71.

#### Perception towards benefit of vaccination

Perception towards benefit of vaccine related four questions were asked by using likert scale from strongly agree to strongly disagree which has five options. After computing the mean score for each constructs, greater than or equal to mean score had positive perception and below means score had negative perception. Cronbach’s Alpha for reliability test was 0.79.

#### Client satisfaction

Satisfaction related ten questions were asked using likert scale. After computing the mean score for each constructs, greater than or equal to mean score were satisfied and below mean score were not satisfied. Cronbach’s Alpha reliability test was 0.76.

### Study variables

#### Dependent variables

Incomplete vaccination.

#### Independent variables

##### Socio-demographic and economic variables

Mother’s age, Place of residence, Educational status of mother or care giver, Educational status of father, Father’s occupation, Family size, Religion, Sex of child, Age of child, Birth order of the child and wealth index.

##### Health service related characteristics of mother or child

ANC attendance, Place of delivery, PNC follow up, TT vaccination of mother, Availability of vaccine, Postponing schedule, Time of travel to health facility, inconvenient time of vaccination.

##### Mather/care giver related variables

Knowledge on schedule and vaccine preventable disease and perception on vaccine contra indication.

### Data collection method and procedure

Data was collected using a structured questionnaire adapted from WHO immunization survey and other literatures^11,13^. Questionnaire includes socio demographic factors, health service related factors, and mother/care giver related factors that affect default from fully immunization. It was originally prepared in English and then translated to local language Afan Oromo then retranslated back to English to check for its consistency.

An interviewer administered semi-structured questionnaire was used to collect the data from care givers of the children’s. Two diploma nurses and four health extension worker was recruited for data collection. Two days training was provided for both supervisors and data collectors on data collection instruments and procedure. Data quality was ensured by giving training for data collectors and strict supervision during data collection. A pretest was conducted on 5% of the sample size in the mettu district before the actual data collection.

### Data quality management

Questionnaires first was prepared in English language and later translated to local language Afan Oromo and then retranslated back by other translator to English to compare the consistency. Before actual data collection, the questionnaire was pre-tested similar population in another district in the Zone by taking 5% of the total sample and necessary modifications were done. Data collectors and supervisors were trained for two days on the study instrument and data collection procedure and ethical issues. During the actual data collection process, supervisors cross checked the data on every day for consistency and completeness. After data collection, each questionnaire was given a unique code by the principal investigator.

### Data Processing and Analysis

Data was checked for completeness, cleaned, coded and, finally entered to Epi-data version 3.1 and analyzed by using SPSS version 23. Descriptive statistics, including frequencies and percentages, was calculated to describe demographic and economic, health service related characteristics of mother/child, and mother or care giver related characteristics. Bivariate analysis, variables with a p-value less than 0.25 was candidate to a multivariable logistic regression. In the multivariable analysis, backward stepwise logistic regression at p-value ≤ 0.05 was used to identify independent determinants of incomplete vaccination. The Hosmer–Lemeshow goodness of-fit checked at p, value 0.86. Multicollinearity between each independent variable was diagnosed using Variance Inflation factor (VIF). As a result, VIF values larger than 10 were thought to be noticeable of collinearity. Finally, the strength association was checked by odds ratio (OR) at 95% CI and p value of less than 0.05.

### Ethical approval and consent to participate

The study followed the Helsinki Declaration of Ethical Principles for Medical Research Involving Human Subjects. Accordingly, ethical approval letter (Ref.no. DPH 169/2022) was received from the Institutional Ethical Review Committee of Colleges of Health Sciences, Mattu University. Then a letter of support was taken from the health offices of the mettu district. After giving detailed information about all ethical components and the study’s aims, written informed consent was obtained from the child caretaker However, verbal consent was obtained from the participants who were unable to read and sign the consent form, considering their poor literacy status to understand and sign the consent form. The data collector loudly read the consent form to each participant and allowed the participant to ask any unclear questions about the form. The participant was given full authority to leave at any moment during the interview. In addition, the participants’ information was kept anonymous to secure confidentiality.

## Results

### Socio-demographic Characteristics

Total of 292 (95 cases and 197 controls) were participated in the study from 297 selected participants with response rate of 98% (96% of cases and 99% of controls). The mean ± SD ages of respondents (mothers/caretakers) and children in both groups (cases and controls) were 29 ± 5.83 years and 17.4 ± 3.1 months, respectively. However, the mean ± standard deviation ages of mothers and carers among cases and controls were 30 ± 6.93 and 28.3 ± 5.07, respectively. Majority of cases were Protestant religion followers 62(65.3%) and 131(66.5%) among controls respectively. Majority of respondents were Rural residence 81(85.3%) among cases and 127 (64.5%) among controls (Table 1).

**Table 1:** Socio-demographic characteristics of caregivers and children under study in Illu Aba Bor, Mettu district, Southern West Ethiopia 2022.

Variables	Category	Cases N (%)	Control N (%)
Primary Care giver	Mother	79 (83.2)	173 (87.8)
	Grand Mother	10 (10.5)	16 (8.1)
	Others	6 (6.3)	9 (4.1)
Age of respondent	< 35 years	42 (44.2)	96 (48.7)
	> = 35 years	101 (51.3)	53 (55.8)
Residence	Rural	81 (85.3)	127 (64.5)
	Urban	14 (14.7)	70 (35.5)
Marital status	Married	75 (78.9)	158 (80.2)
	Divorced	8 (8.4)	20 (10.2)
	Others	12 (12.7)	19 (9.4)
Respondent education	≤ Primary	38 (40.0)	73 (37.1)
	> Primary	57 (60.0)	124 (62.9)
Spouse education	≤ Primary	44 (46.3)	69 (35.0)
	> Primary	51 (53.7)	126 (65.0)
Spouse occupation	Farmer	77 (81.1)	119 (60.4)
	Merchant	8 (8.4)	39 (19.8)
	Gov’t employee	4 (4.2)	21 (10.7)
	Others	89 (6.3)	18 (9.2)
Age of child	12–18 month	45 (47.4)	98 (49.7)
	19–23 month	50 (52.6)	99 (50.3)
Birth order of child	≤ 2nd	48 (50.5)	90 (45.7)
	≥ 3rd	47 (49.5)	107 (54.3)
Family size	≤ 5	52 (54.7)	97 (49.2)
	> 5	43 (45.3)	100 (50.8)
Ethnicity	Agnewa	82 (86.3)	166 (84.3)
	Oromo	4 (4.2)	26 (13.2)
	Others	9 (9.5)	5 (2.5)
Religion	Protestant	62 (65.3)	131 (66.5)
	Muslim	12 (12.6)	33 (16.8)
	Orthodox	13 (13.7)	22 (11.2)
	Others	8 (8.4)	11 (5.6)
Sex of child	Male	43 (45.3)	94 (47.7)
	Female	52 (54.7)	103 (52.3)
Wealth index	Poor	59 (62.1)	94 (47.7)
	Rich	36 (37.9)	103 (52.3)

### Mother/care giver knowledge and perception

Concerning perception towards the benefit vaccination, 64(67.4%) among cases and 157(79.7%) among controls mothers had good perception. Besides, regarding satisfaction with vaccination service, only 35(36.8%) among cases and 75(38.1%) controls mother or care giver were satisfied (Table 2).

**Table 2:** Knowledge, Perception and related factors of care takers living in Illu Aba Bor, Mettu district, South West Ethiopia 2022.

Variables	Category	Cases N (%)	Controls N (%)
Perception on side effect	Favorable	67 (70.5)	130 (66.0)
	Non Favorable	28 (29.5)	67 (34.0)
Perception on benefit	Positive	64 (67.4)	157 (79.7)
	Negative	31 (32.6)	40 (20.3)
Knowledge on VPDs	Good	47 (49.5)	120 (60.9)
	Medium	12 (12.6)	27 (13.7)
	Poor	36 (37.9)	50 (25.4)
Knowledge on schedule	Good	61 (64.2)	132 (67.0)
	Poor	34 (35.8)	65 (33.0)
Client satisfaction	Satisfied	33 (34.7)	85 (43.1)
	Not satisfied	62 (65.3)	112 (56.9)

### Maternal and child health service related risk factors

More than half 54(56.8%) cases and 59(29.9%) controls were postponed their vaccination schedule. In addition to this only 30(31.6%) cases and 128(65.0%) controls mothers had received tetanus vaccination during their pregnancy **(**Table 3).

**Table 3:** Health service utilization of care takers living in Illu Aba Bor, mettu districts, South West Ethiopia, 2022.

Variables	Category	Cases (N%)	Controls N(%)
Time to reach HF	< 30 min	32 (33.7)	103 (52.3)
	> = 30 min	94 (47.7)	63 (66.3)
Antenatal care visit	Yes	61 (64.2)	150 (76.1)
	No	34 (35.8)	47 (23.9)
Place of delivery	Home	49 (51.6)	74 (37.6)
	Health facility	46 (48.4)	123 (62.4)
Postnatal care visit	Yes	41 (43.2)	107 (54.3)
	No	54 (56.8)	90 (45.7)
Poste pond vaccination schedule	Yes	54 (56.8)	59 (29.9)
	No	41 (43.2)	138 (70.1)
TT Vaccination	Yes	30 (31.6)	128 (65.0)
	No	65 (68.4)	69 (35.0)

### Determinants of incomplete penta vaccination

In bivariate analysis residence and wealth index antenatal care visit, place of delivery, attend postnatal care, maternal history of TT vaccination, postponed vaccination schedule, knowledge of vaccine preventable diseases, perception on benefit of vaccination, time taken to reach vaccination site and client satisfaction on immunization service were candidate for multivariable logistic regression at p-value < 0.25. In multivariable logistic regression analysis indicated residence, wealth index; mothers received tetanus vaccination, post pond vaccination schedule and perception toward benefit of vaccination and client satisfaction were significantly associated with incomplete penta vaccination.

Rural residences were 4 times more likely to be incomplete penta vaccine than urban residence [AOR: 3.9; 95% CI; (1.6**–**9.4)]. Additionally family wealth index of poor were more than 3 times more likely to be incomplete vaccine than compared to wealth index of rich (AOR: 3.6; 95% CI; (1.8**–**7.0). Whereas, mothers did not received tetanus vaccination were more than 4 more times likely incomplete vaccine than mothers received tetanus vaccination their children [AOR: 4.3; 95% CI; (2.1**-** 8.6)]. Furthermore, children whose their mothers postponed vaccination schedule were more than 4 times more likely incomplete vaccination than not post ponded vaccination schedule [AOR: 4.6; 95% CI; (2.4**–**8.8)].

Moreover, children whose mother not satisfied on vaccination service were more than 3 times more likely incomplete vaccination than children whose mother or care giver satisfied with immunization service [AOR: 3.7; 95% CI; (1.7**–**7.6)]. Lastly, children whose their mothers had poor perception on benefit of vaccine were more than 2 times more likely to be incomplete vaccination than those mother had good perception towards the benefit of vaccination [AOR: 2.7; 95% CI; (1.2–6.1)](Table 4).

**Table 4:** Bivariate and Multivariable Logistic regression analysis of determinants of incomplete vaccination in Illu Aba Bor, Mettu district, South west Ethiopia 2022.

Variables	Category	Incomplete vaccination		COR (95%CI)	P-value	AOR (95%CI)	P-value
		Case N.(%)	Control N.(%)				
Residence	Rural	81 (85.3)	127 (64.5)	3.2 (1.7,6.0)	0.001	**3.9 (1.6, 9.4)**	**0.02**
	Urban	14 (14.7)	70 (35.5)	1		**1**	
Wealth index	Poor	59 (62.1)	94 (47.7)	1.8 (1.1, 3.0)	0.022	**3.6 (1.8, 7.0)**	**0.01**
	Rich	36 (37.9)	103 (52.3)	1		**1**	
Client satisfaction	Satisfied	33(34.7)	85 (43.1)	1		**1**	
	Not satisfied	62 (65.3)	112 (56.9)	1.4 (0.9,2.3)	0.171	**3.7 (1.7, 7.6)**	**0.01**
Knowledge on VPDs	Good	47 (49.5)	120 (60.9)	1			
	Poor	48 (50.5)	77 (39.1)	1.6 (0.97,2.6)	0.065	1.01 (0.97,2.6)	**0.06**
Perception on benefit	Positive	64 (67.4)	157 (79.7)	1		**1**	
	Negative	31 (32.6)	40 (20.3)	1.9 (1.1,3.3)	0.022	**2.7 (1.2, 6.1)**	**0.017**
Poste pond vaccination schedule	Yes	54 (56.8)	59 (29.9)	3.1 (1.8,5.1)	0.001	**4.6 (2.4, 8.8)**	**0.01**
	No	41 (43.2)	138 (70.1)	1		**1**	
TT Vaccination	Yes	30 (31.6)	128 (65.0)	1		1	
	No	65 (68.4)	69 (35.0)	4.0 (2.4,6.8)	0.001	**4.2 (2.1, 8.7)**	**0.01**

Significant values are in bold.

## Discussion

This study identified that rural residence was 4 times more likely to be incomplete penta vaccination than urban residence. This finding is in line with study conducted in Hawassa, Afghanistan and in Cô te d ’ Ivoire^14–17^ This could be related with disparity of health facility between the communities and might be less mobilization of the community in rural area about vaccination.

Poor wealth index was found to be associated were more than 3 times more likely to be incomplete penta vaccination than wealth index of rich. This finding is comparable with study conducted in Hawassa Zuria district^14,18^. This might be attributable to household with good economic status were able to cover the cost involved during repeated visits like transportation and other related cost. Besides, absence of transportation, low access to media likes radio and TV and poor knowledge due to low educational status.

Women did not receive tetanus vaccination were more than 4 times more likely incomplete vaccine than those received tetanus vaccination their children. This finding is agreed with study conducted in Kenya, Hossana and Jigjiga^19–21^, respectively. The possible justification might be when women had received tetanus vaccination they get good experience and good perception on the benefit of vaccination and also they avoid postponed of vaccine schedule as well as they can fully vaccinate their children. Additionally they also get good information about immunization related knowledge which was obtained from repeated education during tetanus vaccination.

Furthermore, a child who’s their mothers postponed vaccination schedule were more than 4 times more likely incomplete vaccination than not post ponded vaccination schedule. This finding is likeness with the study conducted in South and Lare district Ethiopia^13,22^. In contrast to this finding study conducted in Adibo district shows postpone vaccination schedule was not associated with complete childhood vaccination^5^. The possible justification might be child and caretaker sickness, time of delivery service, forgetting the schedule, failure to inform caretakers about the need of timely vaccination of subsequent doses, absence of some vaccine vial during appointment time special some vaccine which are opened for ten and above children. In the study area, the main difference between cases and controls were forgetting the schedule and absence of some vaccine vial prepared for 10–20 children to give on one time if opened.

Moreover, children whose mother not satisfied on vaccination service were more than 3 times more likely incomplete vaccination than children whose mother or care giver satisfied with immunization service. This support study conducted in Beshanigulgumuz^23^. However, study conducted in Matakel district reveal no association between satisfaction status and incomplete childhood vaccination^10^. This might be more satisfied caretakers might have positive perception towards health workers and more likely encouraged to seek vaccination thereby completing the recommended dose for their child.

Finally children whose their mothers had poor perception on benefit of vaccine were more than 2 times more likely to be incomplete vaccination than those mother had good perception towards the benefit of vaccination . This is consistent with study conducted Ardigona district and Wonago District^22,24^. The possible consistency might be due to mothers or care giver who did not know about benefit of vaccine was not motivated to vaccinate their child.

## Strength and limitation of the study

### Strengths

Being a community-based study as well as the study were used a case control study design and identified some determinants incomplete vaccination in the communities to be the strengths of this study.

### Limitations

Recall bias and social desirability bias may be introduced due to self-reported question even though efforts were done to reduce bias. The readiness of facilities to improve immunization delivery service was not reviewed.

## Conclusion

The study identified that rural residence, Family wealth index of poor; Mother not received tetanus vaccination; postponed vaccination schedule client satisfaction and caretaker perception on benefit of vaccination were identified determinants of incomplete vaccination.

Health information should be given for the community and child caretaker on the benefit of complete vaccination. Community should be encouraged to not post pond vaccine schedule. Pregnant women should be strengthening to receive tetanus toxoid vaccine during pregnancy.

## Data Availability

All data used in this study are available in the manuscript.

## Abbreviations

ANC: Antenatal care
AOR: Adjusted odds ratio
BCG: Bacillus calmette guirine
CI: Confidence interval
COR: Crude odds ratio
DTP: Diphtheria tetanus and pertussis
EPI: Expanded program for immunization
EDHS: Ethiopia demographic and health survey
ETB: Ethiopian birr
GAVI: Global alliance for vaccines and immunization
HIB: Homophiles influenza type b
HEW: Health extension worker
HSTP: Health sector transformation plan
MOH: Ministry of health
OPV: Oral polio vaccine
OR: Odds ratio
PNC: Postnatal care
PCV: Pneumococcal conjugated vaccine
VPD: Vaccine preventable disease
WHO: World health organization
